# Clinical Approach in Prosthetic Treatment With 3D-Printed Implant-Retained Removable Denture: DENTCA System

**DOI:** 10.1155/2024/9886369

**Published:** 2024-10-10

**Authors:** Aleksandar Naydenov, Nikolay Apostolov, Rumen Radev

**Affiliations:** Department of Prosthetic Dental Medicine, Faculty of Dental Medicine, Medical University-Sofia, 1 “St. G. Sofiyski” Blvd. 1431, Sofia, Bulgaria

**Keywords:** denture, implant, removable

## Abstract

The aim of this study is to present a method that optimizes clinical and laboratory workflow in the fabrication of implant-supported removable dentures by combining conventional and digital protocols. A 73-year-old patient came to our clinic for treatment of a completely edentulous lower jaw. Two Neodent Helix GM implants were placed in the canine regions and a removable denture with Locator GM Novaloc retention elements was fabricated. The DENTCA system was used for impressions, border molding, and to determine both occlusovertical dimension (OVD) and reproducible, physiological position of lower jaw (RPPLJ) in a single clinical visit. In result, we have fabricated a removable denture with implant retention in just two appointments. We can conclude that DENTCA system is a reliable method that allows fabrication of implant-retained removable denture in two clinical visits. The registration of the prosthetic field boundaries, OVD and RPPLJ, combined with CAD technologies, represents a contemporary and accurate method. It takes less time, and expenses are reduced both for the dentist and the patient.

## 1. Introduction

It is necessary to precisely record the boundaries of the prosthetic field, occlusovertical dimension (OVD), and reproducible, physiological position of lower jaw (RPPLJ) for optimal aesthetics, function, and phonetic articulation in the fabrication of complete dentures. Various empirical and controversial methods with no scientific backing exist for recording interjaw relations. There is no strict single method to determine interjaw relations.

Such an approach for recording the height of the interjaw relation is the anatomical-physiological method. This method establishes that the interjaw relation is 2–4 mm lower than the physiological rest position of the lower jaw (LJ). Measurements were taken between the reference points subnasale and gnathion or pronasale and gnathion [1]. The average distance between the physiological rest and central occlusion of the LJ in patients with partial or complete edentulism is 2.22 ± 1.37 mm [2]. To achieve greater precision in RPPLJ registration, the clinician must apply a combination of methods [3]. Intraoral graphic recording (IGR) has been used for nearly a century to determine RPPLJ [4].

We use a dynamic principle such as the 3-point system such as the IGR method in which the patient performs targeted movements of the LJ forward, left, and right with continuous contact of the recording screw to a registration plate. This method graphically registers the sagittal angle. It is assumed that during the registration of the position, the joint heads pass through the most posterior, unstrained, involuntary, and most comfortable position in the joint sockets, which is determined as the RPPLJ. The RPPLJ registered by IGR coincides with the apex of the sagittal angle.

Despite accuracy of this method, it is still relatively rarely used in practice due to the complexity of the technique and assembly difficulties of the early versions of the methodology [4].

Complete dentures can also be classified by whether they are fabricated with conventional or digital methods. The first prosthesis made using CAD technologies was described in 1994 [5]. Since then, digital technologies have been continuously improving. In their research, Maniewicz et al. found that the digitally manufactured complete dentures provided retention and fit similar to that of conventionally manufactured bases and can therefore be considered suitable techniques. [6]

## 2. Case Report

The patient, M.M., a 73-year-old male, came to our clinic for treatment of complete edentulism in the LJ. The medical history revealed controlled Stage 2 hypertension, and arthritis was present, too. Medications were Moxogamma 0.3 mg, Alodagra 100 mg, and Profihol Forte. No allergies were reported. Antagonists are a complete removable upper denture. After intraoral examination and CBCT, the following treatment plan was proposed to the patient: placement of two implants in the canine areas (33, 34) with a subsequent plan for an implant-supported removable denture retained via the GM Novaloc system.

The LJ denture is classified as RP4 according to Misch's classification [7]. The implantation followed the ITI classification Type 4 (late implantation), performed in two stages [8].

Neodent GM implants with dimensions of 4 mm × 10 mm, were used. Conventional loading protocol was followed [9]. Three months after implantation, the sulcus formers were placed for a period of 2 weeks. An impression of the prosthetic field was taken at the implant abutment level as well as the determination of the OVD and RPPLJ of the LJ in a single clinical visit using the DENTCA system. The treatment proceeded in two visits with the following steps.

### 2.1. The First Visit

Selection of impression trays for both the upper jaw (UJ) and LJ from the DENTCA system kit.

Selecting the appropriate material for the final impression of the prosthetic field—a silicone, Elite HD putty, and Elite HD light from Zhermack.

**Figure 1:**
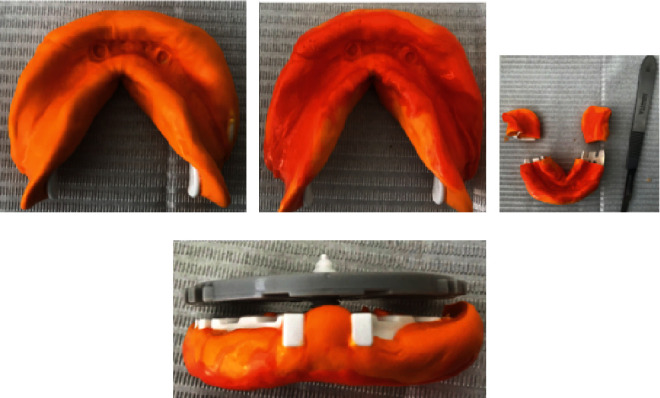
Double-mix impression technique—with A-silicone putty (a) and light body (b). Separating the impression in the tray distally (c). Fixating the registration plate and the impression via the screw (d).

Taking impression of the LJ—2-step double-mix impression technique. First with A-silicone putty consistency (Figure 1), then followed by A-silicone light body (Figure 1).

Separation of distal segments in LJ tray with a scalpel (Figure 1). This separation is necessary because the distal sections are higher than the screw used to determine the OVD, thus posing an obstacle. In our case, it was necessary to reduce the height of the screw, and then the UJ plate was positioned on the screw (Figure 1).

**Figure 2:**
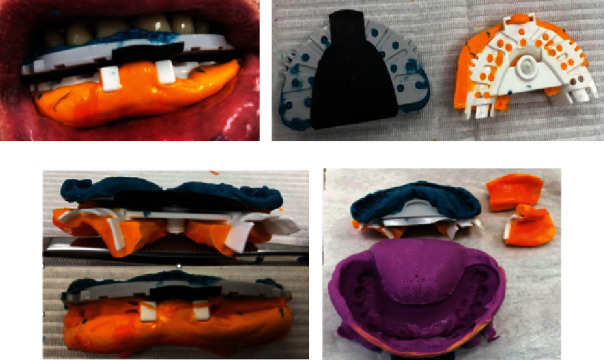
Taking antagonist impression with the special tray used only for RPPLJ and OVD registration (a). After registration the OVD and RPPLJ with EZ-Tracer, an arrow with an angle pointing palatally is depicted on it (b). Bite registration silicone is used to mount intraorally lower jaw tray and the special plate (c). An impression of the antagonist is taken (d).

The impression was taken with the special plate from the antagonists—used only as a key during the determination of the RPPLJ, as well as for adjusting the impression/model of the antagonists (Figure 2).

Then, resting vertical dimension (RVD) was determined by making the patient close his lips one each to other with all the muscles in rest position and after saying the letter “M,, which is a well-known method [10]. The referent points subnasale and gnathion were marked with a pencil and distance between them was measured.

The OVD was assessed by screwing the screw attached to the lower tray in the direction of the upper plate until we reach a height of 2 mm less than the one determined when registering the initial RVD. The correct OVD position was confirmed by calculating if the newly established OVD is in fact 2 mm less than the established RVD.

Attach the EZ-Tracer to the designated surface of the plate, which was previously used for taking impression from antagonists in UJ (Figure 2).

Registration of the RPPLJ with the screw and the specific methodology are described in the system's manual for performing the necessary movements in the LJ by the patient during which the plate must be held statically by the antagonists in the UJ (Figure 2).

After registering the RPPLJ, an arrow with an angle pointing palatally is depicted on the EZ-Tracer (Figure 2). The tip of the angle is the location where the RPPLJ is projected. A hole with minimal dimensions is drilled corresponding this location so that the sharp end of the screw can be statically fixed in it.

After fixating the impressions taken from the UJ and LJ to each other using the screw, a minimal space is revealed between the two trays. This space is fulfilled with silicone for occlusion registration, and after its elastification, the impressions from the UJ and UJ become an inseparable unit. This unit consists of the data of prosthetic field boundaries, OVD, and RPPLJ. The smile line, midline, and canine line are marked on the impression with a marker (Figure 2). For the impression of antagonist teeth, the complete denture in UJ was taken (Figure 2).

**Figure 3:**
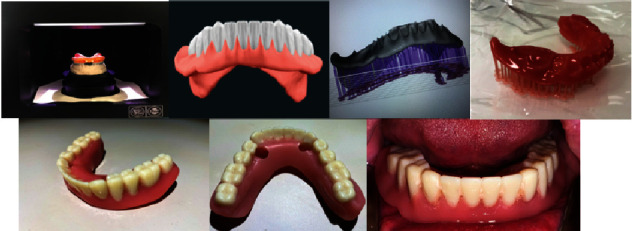
Scanning of the impressions from the lower jaw attached to the gypsum model of the upper jaw. 3D design of both denture bases and artificial teeth on an STL file. Denture base in the 3D printer's software and printed as follows. Attach the teeth to the denture base. Drilled holes in the denture base for intraoral direct attachment of the base to the locators. Intraoral view of the finally attached denture to the implants.

The impressions were scanned with a laboratory 3D scanner (Figure 3).

Files then are imported into the online-based CAD software of DENTCA system. After importing files into the software, 3D modelling and arranging of teeth followed. STL files were created, which can easily be downloaded/sent from the online-based software to an online address (Figure 3).

The STL files are imported in a suitable program for 3D printer preparation. Denture body and the teeth are created in two separate files due to the printer's inability to work with two different types of resins simultaneously. The material used for manufacturing both the body and the teeth is a specialized resin-type methacrylate used for 3D printing—Optiprint by Dentona.

After 3D printing of the teeth and the body of the denture, the denture was processed in an ultrasonic bath with an alcohol solution and further polymerization in a UV oven. Subsequent processing included drilling holes for the housing matrix Novaloc. The teeth and denture body were adhered together with the same resin (Optiprint) and finally polished (Figure 3).

### 2.2. The Second Visit

The denture construction was fixed to the dental implants through the locators and housing matrix directly in the oral cavity using self-curing resin (Figure 3).

### 2.3. Follow-Up Appointments

The first follow-up appointment was 7 days after the placement of the denture. The patient confirmed that he was wearing the denture in this period and that he takes it off only to clean it. There were no complaints regarding to pain, function, and aesthetics. No intraoral abnormalities were observed. The mucosa was healthy and no changes to the denture borders were made.

The next follow-up was scheduled in 6 months. The patient again expressed satisfaction regarding the functionality and aesthetics of the denture. No complaints regarding pain or discomfort were made.

The next follow-up is scheduled in 6 months, 1 year after the placement of the denture.

## 3. Discussion

The DENTCA system allows the fabrication of implant-retained denture in a single clinical visit. On the second visit of the patient, the denture is fixed and adjusted. If a desktop or intraoral 3D scanner is available in the dental office, the dentist is able to fabricate a complete denture within an hour.

The time required for impression taking of the prosthetic field and the registration of the OVD and RPPLJ is about 30 min. This time can be further reduced if the two-step double-mix impression technique is replaced with a single-step impression technique. Two-step impression technique is not mandatory for the success of conventional complete denture fabrication regarding a variety of clinical aspects of denture quality and patients' perceptions of the treatment [11]. Studies carried out by various authors came to the same conclusion that using digital methods greatly reduce the clinical time required [12].

Virtual designing with an online-based software such as the DENTCA system takes about 20 min. The 3D printing process depends on the capabilities of the 3D printer; with the printer we used, it took approximately 4.5 h, with a print accuracy of 50 *μ*m. The final processing after printing takes about 20 min. In summary, a complete denture can be fabricated in less than 5.5 h, of which less than 1 h is active work.

On the contrary, a denture fabricated via conventional methods takes about 5 h of active work by the clinician, spread in several days [13]. The patient is required to visit the clinic multiple times, and the work is highly dependent on the dental technician. This does not include the time spent by the dental technician, which is approximately the same as that of the clinician. A study carried out in 2019 by Resende et al. concluded that the fabrication of conventional removable complete dentures would normally require between five and six clinical visits [14]. Arakawa et al. stated that digitally fabricated removable complete dentures can be considered a viable alternative to conventional removable complete dentures regarding treatment duration, clinical and follow-up visits, adjustments, and maintenance requirements [15].

Creating digital files is easy and convenient to store data about the boundaries of the prosthetic field, OVD, and RPPLJ. If a new denture construction is required, digital files are a significant advantage compared to conventional technology.

Due to the reduced adhesion between 3D-printed teeth and the 3D-printed denture plate compared to the adhesion between the same in the conventional method of fabricating the prosthetic construction, we recommend the whole 3D denture construction to be made as a single printed model, from one type of resin (used for the teeth). After printing, the gingival part of the prosthesis should be impregnated with acrylic paints to reproduce the desired optical characteristics [16]. Furthermore, it should be noted that this method has a significantly reduced impact on the environment compared to conventional methods of fabricating such a denture, which consume much more resources.

The complications and challenges that we encountered with this half-digital method are the following: (1) there are no trays individualized for every single case, so in some cases, trays must be corrected manually. (2) The DENTCA online-based software is not as sophisticated as the special dental CAD software's such as ExoCad and 3Shape.

To overcome these challenges, we recommend that improvements be done in both software and hardware (trays) of the DENTCA system.

In conclusion, the DENTCA system is a reliable method that allows for the fabrication of a completely removable implant-retained denture in just a single clinical visit. The registration of the prosthetic field boundaries, OVD, and RPPLJ in one visit, combined with digital technologies, represents a contemporary and accurate method, which saves time and resources for both the dental practitioner and the patient. We emphasize the need for further case reports or studies to support our findings.

## Data Availability

All data related to the presented case are included within the article.

## Nomenclature

CAD: computer-aided design
CBCT: cone beam computer tomography
IGR: intraoral graphic recording
ITI: International Team for Implantology
LJ: lower jaw
OVD: occlusovertical dimension
RPPLJ: reproducible physiological position of lower jaw
RVD: resting vertical dimension
UJ: upper jaw
